# Targeted and non-targeted proteomics to characterize the parasite proteins of *Echinococcus multilocularis* metacestodes

**DOI:** 10.3389/fcimb.2023.1170763

**Published:** 2023-05-30

**Authors:** Joachim Müller, Matías Preza, Marc Kaethner, Reto Rufener, Sophie Braga, Anne-Christine Uldry, Manfred Heller, Britta Lundström-Stadelmann

**Affiliations:** ^1^ Institute of Parasitology, Vetsuisse Faculty, University of Bern, Bern, Switzerland; ^2^ Graduate School for Cellular and Biomedical Sciences (GCB), University of Bern, Bern, Switzerland; ^3^ Proteomics and Mass Spectrometry Core Facility, Department for BioMedical Research (DBMR), University of Bern, Bern, Switzerland; ^4^ Multidisciplinary Center for Infectious Diseases, University of Bern, Bern, Switzerland

**Keywords:** cestodes, model system, targeted proteomics, transport, echinococcosis, untargeted proteomics, antigen B

## Abstract

The larval stage of the cestode *Echinococcus multilocularis* is the causative agent of alveolar echinococcosis. To investigate the biology of these stages and to test novel compounds, metacestode cultures represent a suitable *in vitro* model system. These metacestodes are vesicles surrounded by an envelope formed by the vesicle tissue (VT), which is formed by the laminated and germinal layer, and filled with vesicle fluid (VF). We analyzed the proteome of VF and VT by liquid chromatography tandem mass spectrometry (LC-MS/MS) and identified a total of 2,954 parasite proteins. The most abundant protein in VT was the expressed conserved protein encoded by EmuJ_000412500, followed by the antigen B subunit AgB8/3a encoded by EmuJ_000381500 and Endophilin B1 (protein p29). In VF, the pattern was different and dominated by AgB subunits. The most abundant protein was the AgB8/3a subunit followed by three other AgB subunits. In total, the AgB subunits detected in VF represented 62.1% of the parasite proteins. In culture media (CM), 63 *E. multilocularis* proteins were detected, of which AgB subunits made up 93.7% of the detected parasite proteins. All AgB subunits detected in VF (encoded by EmuJ_000381100–700, corresponding to AgB8/2, AgB8/1, AgB8/4, AgB8/3a, AgB8/3b, and AgB8/3c) were also found in CM, except the subunit encoded by EmuJ_000381800 (AgB8/5) that was very rare in VF and not detected in CM. The relative abundance of the AgB subunits in VF and CM followed the same pattern. In VT, only the subunits EmuJ_000381500 (AgB8/3a) and EmuJ_000381200 (AgB8/1) were detected among the 20 most abundant proteins. To see whether this pattern was specific to VF from *in vitro* cultured metacestodes, we analyzed the proteome of VF from metacestodes grown in a mouse model. Here, the AgB subunits encoded by EmuJ_000381100–700 constituted the most abundant proteins, namely, 81.9% of total protein, with the same order of abundance as *in vitro*. Immunofluorescence on metacestodes showed that AgB is co-localized to calcareous corpuscles of *E. multilocularis*. Using targeted proteomics with HA-tagged EmuJ_000381200 (AgB8/1) and EmuJ_000381100 (AgB8/2), we could show that uptake of AgB subunits from CM into VF occurs within hours.

## 1 Introduction

The cestode *Echinococcus multilocularis* (small fox tapeworm) and the closely related *E. granulosus sensu lato* cause the diseases alveolar and cystic echinococcosis, respectively (Woolsey and Miller, 2021). Alveolar echinococcosis (AE) is endemic and emerging in the Northern hemisphere (Deplazes et al., 2017) including regions in Europe (Oksanen et al., 2016), North America (Cerda et al., 2018; Santa et al., 2021), and Asia (Cadavid Restrepo et al., 2018). The annual estimated number of new human infections is 17,400, most of them occurring in China (Casulli et al., 2019), but owing to disease severity and lack of curative drug treatment, it is ranked highest among foodborne parasitic zoonoses in Europe (Bouwknegt et al., 2018). Adult *E. multilocularis* worms colonize the small intestine of definitive hosts (foxes and other canids) and release eggs into the environment *via* the feces of these hosts. Ingestion of infective eggs by intermediate hosts (small rodents and other mammals) leads to AE. Besides rodents, there is a wide range of accidental hosts such as humans, dogs, captive monkeys, and beavers that can get infected by the parasite and develop AE (Thompson, 2017). Upon oral infection with eggs, oncosphere larvae are released in the small intestine of intermediate and accidental hosts, and reach the liver where they transform into multi-vesicular metacestodes. These undergo slow, but potentially unlimited proliferation, similarly to a malignant tumor, and they infiltrate the surrounding tissue, metastasize, and cause chronic AE that is lethal if untreated (Thompson, 2017; Casulli et al., 2019). Treatment options are limited. Radical surgical resection in combination with benzimidazole drug treatment is the only curative option (Kern et al., 2017). Emerging numbers of cases and the lack of curative drug treatment foster the need for new and better drug treatment options (Hemphill and Müller, 2009; Hemphill et al., 2014; Lundström-Stadelmann et al., 2019).

For the development of novel anti-echinococcal therapies, the key of success resides in the understanding of the biology of the disease-causing stage of *Echinococcus*, the metacestode. Since *in vitro* culture systems exist for *E. multilocularis*, this parasite may be considered as a model for other cestodes (Hemphill and Lundstroem-Stadelmann, 2021). In particular, the host–parasite interplay can be studied using this system *in vitro* as well as in *in vivo* mouse models that represent natural hosts of the parasite (Brehm and Koziol, 2017).

Metacestodes consist of an acellular, highly glycosylated carbohydrate-rich laminated layer (LL) surrounding the cellular germinal layer (GL) with the syncytial tegument in between the GL and the LL. Together, these layers comprise the vesicle tissue (VT). The syncytial tegument forms the main barrier for host molecules and it is the most outer living part of the parasite with microtriches protruding into the LL and subtegumentary cytons into the GL (Gottstein and Hemphill, 2008). The GL consists of muscle cells, nerve cells, glycogen storage cells, sub-tegumentary cytons, connective tissue, and undifferentiated stem cells (Brehm and Koziol, 2017; Thompson, 2017). Metacestodes are filled with vesicle fluid (VF) containing metabolites such as di-carbon organic compounds and amino acids and proteins (Ritler et al., 2019).

The protein patterns of VF have been investigated mainly with respect to *E. granulosus s.l.* hydatid fluid obtained from animal or human patients (Aziz et al., 2011; Juyi et al., 2013; Ahn et al., 2015; Santos et al., 2016). These studies have led to the identification of *Echinococcus*-specific antigen B (AgB) subunits, lipid and iron binding proteins, membrane proteins, and proteins involved in the energy metabolism. AgB is a hydrophobic ligand binding protein (HLBP) that is composed of different subunits of 8 kDa each. The function of AgB has not been completely elucidated: In *E. granulosus s.l.*, a cluster of seven gene loci including one copy each for EgAgB8/1, EgAgB8/2, EgAgB8/4, and EgAgB8/5 and three slightly differing copies for EgAgB8/3 are described (Olson et al., 2012). Also for *E. multilocularis*, the AgB cluster contains seven gene loci with one copy of EmAgB8/1, EmAgB8/2, EmAgB8/4, and EmAgB8/5, as well as two identical copies of EmAgB8/3 and a slightly changed form of EmAgB8/3 (Brehm, 2010; Olson et al., 2012). In some studies, the three isoforms of EmAgB8/3 are discriminated as forms a, b, and c (Ahn et al., 2017b). Because of their sequence similarity, AgB subunits form two phylogenetic groups: AgB8/1, AgB8/3 and AgB8/5, and AgB8/2 and AgB8/4 (Muzulin et al., 2008). AgB subunits assemble to oligomers (Monteiro et al., 2012), which are capable of binding lipids and can therefore be considered as apolipoproteins (Obal et al., 2012; Silva-Alvarez et al., 2015b). AgB is, together with antigen 5, the best-characterized antigen of the *E. granulosus s.l.* hydatid fluid, and because of its high antigenicity, it is used for serodiagnosis of echinococcosis (Siles-Lucas et al., 2017). Some studies have highlighted a potential role of AgB of *E. granulosus s.l.* in immunomodulation (Rigano et al., 2001; Siracusano et al., 2008b; Silva-Alvarez et al., 2016). More detailed studies in *E. granulosus s.l.* are hampered by the fact that—contrary to *E. multilocularis*—defined *in vitro* culture systems for metacestodes do not exist yet. Moreover, studies of *E. multilocularis* VF have revealed the existence of extracellular vesicles (Zheng et al., 2017) and proteins from the VF were considered as biomarkers for viability and thus therapeutic success (Ahn et al., 2017c; Valot et al., 2017).

In order to have a tool to investigate not only protein patterns but also proteome dynamics, a standardized *in vitro* system is paramount. In a previous study, we have established such a system for metabolomic studies (Ritler et al., 2019).

In the present study, we used this system of axenically grown *in vitro* metacestodes and employed shotgun proteomics to compare the protein patterns of VF and VT and to analyze the proteins found in the corresponding *in vitro* culture medium. To define the VF in more detail, we compared the pattern of VF from *in vitro* grown metacestodes to VF harvested from metacestodes grown in a mouse model. We then focused on AgB isoforms that are most prominent in VF and experimentally investigated the localization of AgB by whole-mount immunofluorescence within the metacestode and the re-uptake of AgB from culture medium into the metacestode.

## 2 Materials and methods

### 2.1 Chemicals

If not mentioned otherwise, all chemicals used were purchased form Sigma (St. Louis, MO, USA). Cell culture media and fetal bovine serum (FBS) were from Bioswisstec (Schaffhausen, Switzerland).

### 2.2 Experimental design of proteomic samples

The *E. multilocularis* metacestode *in vitro* growth medium was prepared as described in Ritler et al. (2019). In short, Dulbecco’s Modified Essential Medium (DMEM) including 0.1% fetal calf serum was preconditioned with Reuber rat hepatoma cells (RH) for subsequent *E. multilocularis* metacestode vesicle incubation. *E. multilocularis* isolate H95 metacestode vesicles grown *in vitro* for 12 weeks (between 2 and 4 mm in diameter) were used for the interaction study. Metacestode vesicle integrity was visually confirmed during the experiment and before collection of samples. In a first experiment, VT and VF were harvested for subsequent analyses by liquid chromatography tandem mass spectrometry (LC-MS/MS) after 72 h of incubation. In addition, culture medium (CM) from the same setup that was incubated with *E. multilocularis* metacestodes for 72 h was analyzed by LC-MS/MS in a second experiment. In a third experiment, VF samples of experimentally infected BALB/c mice were analyzed *ex vivo.* For each experiment, five independent biological replica were analyzed.

### 2.3 Sample preparation

The metacestode CM of the above-described setup was collected, centrifuged for 10 min at 500 × *g* and 4°C, and supernatants were immediately stored at −80°C for subsequent protein analysis. VT and VF were extracted by washing metacestode vesicles three times in 4°C NaCl (0.9%) and breaking them with a 1-ml pipette tip followed by centrifugation at 9,000 × *g* for 20 min at 4°C. The supernatant (VF) was removed and was centrifuged again at 12,000 × *g* for 20 min at 4°C. The remaining metacestode tissue in the pellet was washed with NaCl (0.9%, 4°C) and centrifuged again as above. The VT pellet and VF supernatant were stored at −80°C for subsequent protein analysis by LC-MS/MS.

### 2.4 Parasite maintenance in BALB/c mice and *ex vivo* sample preparation

Animals for parasite maintenance were purchased from Charles River Laboratories (Sulzheim, Germany) and used for parasite maintenance after 2 weeks of acclimatization. BALB/c mice were maintained in a 12-h light/dark cycle, at a controlled temperature of 21°C–23°C, and at a relative humidity of 45%–55%. Food and water were provided *ad libitum*. All animals were treated in compliance with the Swiss Federal Protection of Animals Act (TSchV, SR455) and were approved by the Animal Welfare Committee of the canton of Bern under license number BE30/19. *E. multilocularis* metacestodes (isolate H95) were grown in intraperitoneally infected mice (BALB/c). The mice were euthanized with CO_2_ after 3–4 months of parasite growth. VF of five independently infected animals was aseptically removed by syringe and stored at −80°C until further analysis.

### 2.5 Liquid chromatography tandem mass spectrometry

An aliquot of 5 μl of StrataClean™ resin slurry (Agilent Technologies) was added per 1 ml of CM or VF for the extraction of proteins. The medium was incubated at room temperature by rotation for 15 min on a Ferris wheel. Beads were spun down for 2 min at 230 × *g*. The medium was transferred to a new tube and protein extraction was repeated once more with a fresh aliquot of resin.

The VT samples were resuspended in 250 µl of Lysis buffer (8 M Urea, 100 mM Tris, and Roche protease inhibitor cocktail) and sonicated on ice with 10-s intervals for two cycles. The protein concentration was determined by BCA assay. An aliquot corresponding 20 µl of VT lysate was processed by the same SDS-PAGE strategy as resin-enriched samples (see below). Before loading on the gel, proteins were reduced by the addition of 4 μl of 40 mM DTT for 30 min at 37°C, alkylated with 2 μl of 0.5 M iodoacetamide for 30 min at 37°C in the dark, followed by quenching of iodoacetamide by the addition of 8 μl of 0.1 M DTT.

The resin supernatants of the VF and CM samples were then discarded, and proteins of each resin pellet were extracted with 15 μl of SDS-PAGE sample buffer (62.5 mM Tris-HCl, pH 6.8, 2% SDS, 25% glycerol, 0.01% bromophenol blue) by boiling for 5 min at 95°C. The extracts of VF, VT, and CM were loaded on a 12.5% SDS-PAGE and proteins were separated for about 1 cm. After Coomassie staining and destaining, the lane was cut into five horizontal slices. Proteins were in-gel digested as described elsewhere (Gunasekera et al., 2012). The digests were analyzed by liquid chromatography LC/MS-MS (Easy1000 nanoLC coupled to a QExactive classic or HF mass spectrometer, ThermoFisher Scientific) with one injection of 5-μl digests. Peptides were trapped on a C18 PepMap100 precolumn (5 μm, 100 Å, 300 μm × 5 mm, ThermoFisher Scientific, Reinach, Switzerland) and separated by backflush on a C18 column (3 μm, 100 A°C, 75 μm × 15 cm, Nikkyo Technos, Tokyo, Japan) by applying a 40-min gradient of 5% acetonitrile to 40% in water, 0.1% formic acid. The flow rate on the QExactive classic setup was 300 nl/min. The Full Scan method was set with a mass range of 360–1,400 *m*/*z*, a resolution at 70,000 with an automatic gain control (AGC) target of 10^6^, and a maximum ion injection time of 50 ms. A data-dependent method for the 10 most intense precursor ion fragmentations was applied with the following settings: dynamic exclusion time of 20 s, resolution of 17,500, AGC of 10^5^, maximum ion time of 110 ms, isolation mass window of 2 *m*/*z*, normalized collision energy of 27%, under fill ratio 1%, charge exclusion of unassigned and 1+ ions, and peptide match preferred, respectively. The QExactive HF method was set with a column flow rate of 350 nl/min. Peptides of *m*/*z* 400–1400 were detected at a resolution of 60,000 applying an AGC target of 10^6^ and a maximum ion injection time of 50 ms. A top 15 data-dependent method for precursor ion fragmentation with a stepped 27% normalized collision energy was applied with the following settings: precursor isolation width of 1.6 *m*/*z*, a resolution of 15,000, an AGC of 10^5^ with a minimum target of 10^3^, a maximum ion time of 110 ms, charge exclusion of unassigned and 1+ ions, peptide match on, and a dynamic exclusion for 20 s, respectively.

LC-MS/MS data were processed with MaxQuant (V 1.5.4.1 or 1.6.14.0) using default orbitrap settings for peak detection, trypsin cleavage disregarding the proline rule, allowing up to three missed cleavages, variable oxidation on methionine, acetylation of protein N-termini, and deamidation of asparagine and glutamine, with strict carbamidomethylation of cysteines, respectively. Match between runs was used within each sample group with a retention time window of 0.7 min. The fragment spectra were interpreted using the WormBase ParaSite version 15 (WBPS15) of *E. multilocularis*. Protein identifications were accepted only if at least two razor peptides at a 1% false discovery rate (FDR) cutoff were identified. The mass spectrometry proteomics data have been deposited to the ProteomeXchange Consortium *via* the PRIDE (Perez-Riverol et al., 2022) partner repository with the dataset identifier PXD040274.

### 2.6 AgB sequence analyses

**Figure 1:**
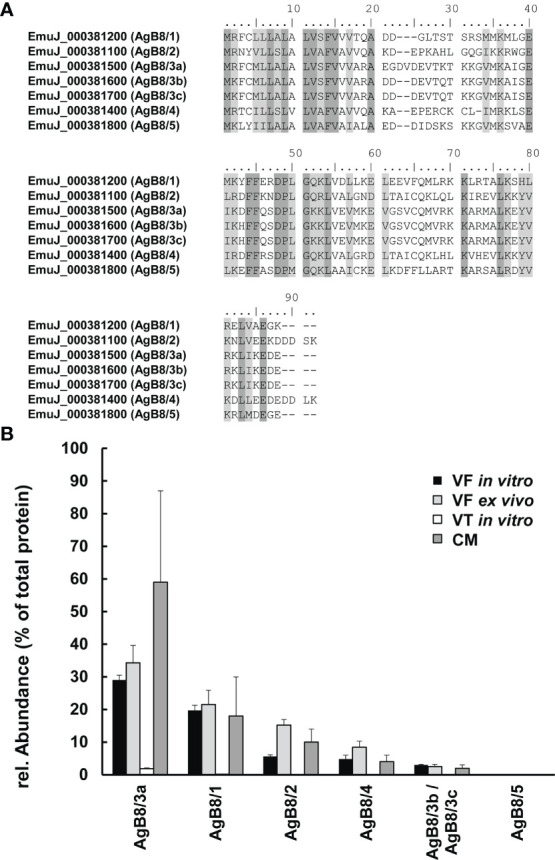
Antigen B (AgB) subunits in *E multilocularis*. **(A)** Alignments of polypeptide sequences of subunits encoded by the ORFs EmuJ_000381100, 200, 400, 500, 700, and 800 without signal peptides. Conserved amino acids are shown in dark gray; similar ones are shown in light gray. Annotations to AgB subunits AgB8/1–AgB8/5 are given in parentheses. **(B)** Relative abundances of these subunits in vesicle fluid (VF *in vitro*), vesicle tissue (VT), and culture medium (CM) of cultured metacestodes, as well as VF from metacestodes *ex vivo* (VF *ex vivo*). Note that EmuJ_000381600 and EmuJ_000381700 cannot be distinguished. Mean values ± standard deviations of five biological replicates are shown.

For the different AgB isoforms, we applied the previously published annotations (Ahn et al., 2017b) for the gene sequences from WBPS15 of *E. multilocularis* (see **Figure 1A**). Sequence alignments were performed using Clustal (www.expasy.org) with default settings. Identical and similar amino acids were identified and highlighted.

### 2.7 Immunofluorescence

Synthetic HA-tagged AgB subunits (AgB8/1–AgB8/5) and respective affinity-purified immune sera were purchased from Pepmic Co. Ltd (Suzhou, China). Polypeptide sequences are given in **Table S1**. In short, rabbits were immunized with synthetic polypeptides eight times in total, and sera were affinity purified against the respective polypeptides. Reactivity was confirmed by ELISA at 1:32,000 dilution with the affinity-purified antisera.

The affinity-purified immune sera against all five AgB polypeptides were tested on pure polypeptides (AgB8/1–AgB8/5) for reactivity and cross-reactivity by dot blot. Polypeptides were diluted in 1:4 steps from 125 to 2 ng in PBS and spotted in 2-µl drops onto nitrocellulose. The polypeptides, against which cross-reactivity was tested, were spotted at the highest amount of 125 ng only. Moreover, VF was spotted in serial dilutions from pure to 1:256 diluted. The blots were blocked in PBS/Tween 0.1%/NaN_3_ 0.05%/milk powder 3% and the antiserum was diluted (1/2,000) in PBS/Tween 0.1%/NaN_3_ 0.05%/milk powder 3%. The blots were incubated overnight at 4°C, washed three times in PBS/Tween, and decorated with goat-anti-rabbit-alkaline phosphatase (1/2,000) in PBS/Tween. After 1-h incubation at room temperature, blots were washed twice with PBS/Tween, followed by PBS and water, and incubated in 0.1 M Tris-Cl^-^, pH 9.5, containing 0.1 M NaCl, 5 mM MgCl_2_, and 3 ml/L NBT/BCIP color reagent (Roche, Mannheim, Germany) until the appearance of blue colored dots.

Whole-mount immunofluorescence was performed on *in vitro* grown, host-cell free *E. multilocularis* metacestodes using the five affinity-purified antisera. The previously described protocol was applied (Koziol et al., 2013) with slight modifications. Briefly, metacestodes were fixed with 4% paraformaldehyde (PFA) in PBS, washed with PBS with 0.3% Triton X-100 (PBS-T), permeabilized for 20 min in PBS with 1% sodium dodecyl sulfate (SDS) for metacestode vesicles, and re-fixated with 4% PFA in PBS. To diminish the autofluorescence, samples were washed three times with PBS and 25 mM of sodium borohydride in PBS was added for 10 min, before washing again with PBS three times. The samples were then washed with PBS/Tween and blocked in PBS/Tween with 3% bovine serum albumin (BSA) and 5% sheep serum. Thereafter, primary antiserum incubation (dilution 1/400 in in PBS/Tween with 3% BSA) was conducted overnight at 4°C. After extensive washing with PBS-T, the samples were incubated overnight with the conjugated secondary antibody (anti-rabbit antibody FITC 1/500 diluted in PBS-T with 3% BSA) at 4°C. Finally, extensive washing with PBS/Tween was performed and a co-staining was made with 4’,6-diamidino-2-phenylindole (DAPI) to visualize nuclei and rhodamine-phalloidin (PHDR1, cytoskeleton) to visualize actin filaments (Adams and Pringle, 1991; Chazotte, 2010). Negative controls were stained in the same way with preimmune sera applied as primary antibodies. Pictures were taken with a confocal laser scanning microscope (Olympus FV3000) and analyzed with the free code software ImageJ version 1.53t in its enhanced version FIJI (Schindelin et al., 2012).

### 2.8 Uptake of HA-tagged AgB

To investigate whether excreted AgB may be taken up by *E. multilocularis* metacestode vesicles, an uptake experiment was performed based on HA-tagged AgB8 polypeptides. For calibration, various amounts of the AgB8/1-HA and AgB8/2-HA polypeptides were spiked into previously harvested VF. For the uptake experiments, AgB8/1-HA or AgB8/2-HA (50 mg/L) was added to preconditioned CM (Ritler et al., 2019) containing 11-week-old metacestode vesicles of 2 to 5 mm in diameter. At various time points from 0 to 24 h, medium samples were taken and vesicles were carefully removed from the medium by sieving through a 40-µm cell strainer (Corning, Corning, NY) and washed once with 30 ml of PBS. VF was then harvested by a syringe (27G needle), pooled from several vesicles (at least three) to obtain a minimum of 50 µl of total volume and stored at −20°C until further processing. The calibration curves were repeated twice for each peptide. The uptake experiment with AgB8/1-HA was performed as a pre-experiment once with independently incubated metacestode vesicles for each time point. For AgB8/2-HA, the experiment was in addition repeated three times independently, and average values and standard errors are given for each concentration and time point.

### 2.9 MS analysis of tagged AgB samples

A volume of 5 µl of VF was diluted with 15 µl of 8 M Urea before reduction by adding 1/10 volume of 0.1 M DTT/100 mM Tris-HCl, pH 8, and incubation for 30 min at 37°C, followed by alkylation (1/10 vol. of 0.5 M iodoacetamide/100 mM Tris-HCl, pH 8, solution, 30 min incubation in the dark) and quenching of iodoacetamide with 4/10 volume of the DTT solution. The samples were then diluted to 4 M by addition of 20 mM Tris-HCl, pH 8.0/2 mM calcium dichloride prior to Lys C (1 µl at 0.1 µg/µl) digestion for 2 h at 37°C, diluted to 1.6 M Urea followed by trypsin (1 µl at 0.1 µg/µl) digestion overnight at room temperature. Digestions were stopped by adding 1/20 volume of 20% (v/v) trifluoroacetic acid (TFA) and transferred into an HPLC vial for LC-MS/MS.

The digests were analyzed on the same QExactive HF mass spectrometer setup as described above with two injections of 5-μl or 8-µl digests for the medium or vesicle samples, respectively, separating peptides with a 60-min gradient of 5% acetonitrile to 40% in water and 0.1% formic acid, at a flow rate of 350 nl/min. A PRM (parallel reaction monitoring) approach was used with inclusion list of AgB8/1-HA polypeptides, set with a resolution at 30,000, an AGC target of 2E05, a maximum ion injection time of 130 ms, an HCD collision energy to 27, under fill ratio 1%, charge exclusion of unassigned and 1+ ions, and peptide match preferred, respectively.

The results were processed with Skyline Software v21.1.0.146 at the MS2 fragment level and the total signal area of the peptide (y and b) transitions was exported into an Excel file. The mass spectrometry proteomics data have been deposited to the ProteomeXchange Consortium *via* the PRIDE (Perez-Riverol et al., 2022) partner repository with the dataset identifier PXD040274.

## 3 Results

### 3.1 Non-targeted proteomic analysis of *E. multilocularis* metacestodes and culture medium

To study the parasite proteome of standardized *E. multiloculari*s metacestodes, we compared the parasite proteomes of VT and VF from *in vitro* cultures. Then, the *E. multilocularis* proteins were identified in corresponding CM, and finally focus was laid on VF obtained from an *E. multilocularis* mouse model, thus designated as VF *ex vivo*. The total numbers of identified peptides and proteins are listed in **Table 1**.

**Table 1:** Summary of protein quantification data.

Study	VF/VT *in vitro*	CM *in vitro*	VF *ex vivo*
**Unique peptides**	43,276	428	10,251
**Non-redundant proteins**	2,954	63	926
**Complete dataset**	**Table S2**	**Table S3**	**Table S4**
**Top 20 proteins**	**Tables 2** and **3**	**Table 4**	**Table 5**

To characterize the proteome of *E. multilocularis* metacestodes, three studies were performed as described in Materials and Methods. VF, vesicle fluid; VT, vesicle tissue: CM, culture medium.

In the study focused on the identification of *E. multilocularis* proteins in VF and VT, 43,276 unique peptides matching 2,954 parasite proteins were identified in total (**Table S2**). The most abundant protein in VT was the expressed conserved protein encoded by EmuJ_000412500, a 124-amino-acid protein with a signal peptide at its N-terminus. This protein was followed by the AgB8/3a subunit encoded by EmuJ_000381500 and the Endophilin B1, also annotated as protein p29. The protein composition of VT was balanced with respect to the relative abundance of proteins, with the most abundant one representing 3% of total proteins and the 20th most abundant protein, parafibromin, representing 0.9% of total proteins (**Table 2**). In VF, the pattern was completely different and dominated by AgB subunits. The most abundant protein was the AgB8/3a subunit encoded by EmuJ_000381500 followed by three other AgB subunits. EmuJ_000381800 (AgB8/5) was also detected, but at a very low abundance (rank 1291). In total, the AgB subunits detected in VF represented 62.1% of total VF proteins. Nineteen amino acids are identical between all AgB subunits, and 21 are similar (**Figure 1A**). The most abundant “non-antigen B protein” was the expressed protein encoded by EmuJ_000415200 with 3% of total proteins, and the 20th most abundant protein, the immunogenic protein ts11 encoded by EmuJ_000543800, represented 0.5% of total proteins (**Table 3**; **Figure 1B**).

**Table 2:** Top 20 *E. multilocularis* proteins in vesicle tissue (VT).

Accession No.	Annotation	Relative abundance (% of total)			
EmuJ_000412500	Expressed conserved protein	3.0	±	0.2	
EmuJ_000381500	Tapeworm specific antigen B (AgB8/3a)	1.9	±	0.3	
EmuJ_000550800	Endophilin B1 (protein p29)	1.9	±	0.0	
EmuJ_000905600	Cytochrome B5	1.8	±	0.1	
EmuJ_000292700	Phosphoenolpyruvate carboxykinase	1.8	±	0.1	
EmuJ_000254600	Glyceraldehyde-3-phosphate dehydrogenase	1.7	±	0.3	
EmuJ_000920600	Myosin heavy chain	1.6	±	0.1	
EmuJ_000703300	Hydroxyacylglutathione hydrolase	1.6	±	0.1	
EmuJ_000122100	Profilin	1.5	±	0.2	
EmuJ_000941100	Dynein light chain	1.5	±	0.3	
EmuJ_000372400	Tegumental protein	1.3	±	0.3	
EmuJ_000417100	Cytosolic malate dehydrogenase	1.2	±	0.1	
EmuJ_000683300	Tetratricopeptide repeat protein 4	1.2	±	0.2	
EmuJ_002165500	Histone H2A	1.2	±	0.1	
EmuJ_001168600	von Willebrand factor A domain containing protein	1.1	±	0.1	
EmuJ_000514200	Enolase	1.1	±	0.1	
EmuJ_000791700	U4:U6 small nuclear ribonucleoprotein Prp4	1.0	±	0.1	
EmuJ_001028500	Lysosomal Pro X carboxypeptidase	1.0	±	0.1	
EmuJ_001068500	ENTH VHS domain containing protein	0.9	±	0.0	
EmuJ_001032250	Parafibromin	0.9	±	0.1	

The complete dataset is presented in **Table S2**. Mean values ± standard deviations correspond to five biological replicates.

**Table 3:** Top 20 *E. multilocularis* proteins in vesicle fluid (VF) *in vitro*.

Accession No.	Annotation	Relative abundance (% of total)			
EmuJ_000381500	Tapeworm specific antigen B (AgB8/3a)	29.0	±	1.5	
EmuJ_000381200	Tapeworm specific antigen B (AgB8/1)	19.7	±	1.6	
EmuJ_000381100	Tapeworm specific antigen B (AgB8/2)	5.6	±	0.5	
EmuJ_000381400	Tapeworm specific antigen B (AgB8/4)	4.8	±	1.2	
EmuJ_000415200	Expressed protein	3.7	±	0.7	
EmuJ_000849600	SET domain containing protein 3	3.6	±	0.2	
EmuJ_000381600/700	Tapeworm specific antigen B (AgB8/3b; AgB8/3c)	3.0	±	0.2	
EmuJ_000682900	Initiation factor eif gamma	1.3	±	0.4	
EmuJ_000908900	Expressed conserved protein	1.1	±	0.2	
EmuJ_000641100	Alpha 2 macroglobulin	1.1	±	0.1	
EmuJ_000315600	Ferritin	1.0	±	0.2	
EmuJ_000292700	Phosphoenolpyruvate carboxykinase	1.0	±	0.3	
EmuJ_000905600	Cytochrome B5	1.0	±	0.2	
EmuJ_000085400	Mastin	0.9	±	0.3	
EmuJ_000417100	Cytosolic malate dehydrogenase	0.8	±	0.1	
EmuJ_000184900	Glycoprotein antigen 5	0.7	±	0.1	
EmuJ_000254600	Glyceraldehyde-3-phosphate dehydrogenase	0.7	±	0.1	
EmuJ_000703300	Hydroxyacylglutathione hydrolase	0.7	±	0.1	
EmuJ_000514200	Enolase	0.5	±	0.2	
EmuJ_000543800	Immunogenic protein ts11	0.5	±	0.0	

The complete dataset is presented in **Table S2**. Mean values ± standard deviations correspond to five biological replicates.

The predominance of AgB subunits in VF prompted us to investigate in a second study whether these subunits were excreted to the CM. In CM, 63 non-redundant *E. multilocularis* proteins were detected (**Table 1**; **Table S3**). The most abundant protein was the AgB8/3a subunit encoded by EmuJ_000381500, representing 59.4% of total *E. multilocularis* proteins (**Table 4**). All AgB subunits detected in VF (encoded by EmuJ_000381100–700) were also found in CM. The subunit encoded by EmuJ_000381800 (AgB8/5) was not detected within the top 20 proteins. The relative abundance of the subunits in VF and CM followed the same pattern: EmuJ_000381500 > 200 > 100 > 400 > 600/700 (e.g., AgB8/3a > AgB8/1 > AgB8/2 > AgB8/4 > AgB8/3b;3c). Note that EmuJ_000381600 (AgB8/3b) and EmuJ_000381700 (AgB8/3c) cannot be distinguished, because polypeptide sequences are identical.

**Table 4:** Top 20 *E. multilocularis* proteins in culture medium (CM).

Accession No.	Annotation	Relative abundance (% of total)			
EmuJ_000381500	Tapeworm specific antigen B (AgB8/3a)	59.4	±	27.5	
EmuJ_000381200	Tapeworm specific antigen B (AgB8/1)	18.4	±	12.2	
EmuJ_000381100	Tapeworm specific antigen B (AgB8/2)	9.9	±	3.7	
EmuJ_000381400	Tapeworm specific antigen B (AgB8/4)	4.4	±	1.8	
EmuJ_000381600/700	Tapeworm specific antigen B (AgB8/3b; AgB8/3c)	1.6	±	0.9	
EmuJ_000385500	Histone H4	1.4	±	0.4	
EmuJ_000905600	Fructose-bisphosphate aldolase	0.9	±	0.4	
EmuJ_000641100	Alpha 2 macroglobulin	0.7	±	0.2	
EmuJ_001077100	Tetraspanin	0.4	±	0.3	
EmuJ_000908900	N acetylated alpha linked acidic dipeptidase 2	0.4	±	0.2	
EmuJ_000315600	Ferritin	0.3	±	0.3	
EmuJ_000407200	Actin cytoplasmic A3	0.2	±	0.0	
EmuJ_000292700	Phosphoenolpyruvate carboxykinase	0.2	±	0.1	
EmuJ_000701800	Basement membrane specific heparan sulfate	0.2	±	0.1	
EmuJ_000682900	Niemann Pick C2 protein	0.2	±	0.3	
EmuJ_000538300	Glutathione S-transferase	0.2	±	0.1	
EmuJ_000849600	Proteinase inhibitor I25 cystatin	0.2	±	0.1	
EmuJ_000724500	Expressed protein	0.1	±	0.1	
EmuJ_000550800	Endophilin B1	0.1	±	0.1	
EmuJ_000122100	Profilin	0.1	±	0.0	

The complete dataset is presented in **Table S3**. Mean values ± standard deviations correspond to five biological replicates.

To see whether this pattern was specific to VF from *in vitro* cultured metacestodes, we analyzed the proteome of VF *ex vivo*, thus from metacestodes grown in a mouse model. A total of 926 parasite proteins were detected (**Table 5**; **Table S4**). The five AgB subunits encoded by EmuJ_000381100–700 constituted the most abundant parasite proteins, namely, 81.9% of total protein, with the same order as *in vitro* and the subunit encoded by EmuJ_000381500 (AgB8/3a) being the most abundant, and the one encoded by EmuJ_000381800 (AgB8/5) being the least abundant subunit (**Table 5**; **Figure 1B**).

**Table 5:** Top 20 *E. multilocularis* proteins in vesicle fluid (VF) *ex vivo*.

Accession No.	Annotation	Relative abundance (% of total)			
EmuJ_000381500	Tapeworm specific antigen B (AgB8/3a)	34.3	±	5.3	
EmuJ_000381200	Tapeworm specific antigen B (AgB8/1)	21.5	±	4.4	
EmuJ_000381100	Tapeworm specific antigen B (AgB8/2)	15.2	±	1.7	
EmuJ_000381400	Tapeworm specific antigen B (AgB8/4)	8.4	±	1.9	
EmuJ_000381600/700	Tapeworm specific antigen B (AgB8/3b; AgB8/3c)	2.5	±	0.7	
EmuJ_000415200	Expressed protein	2.5	±	1.4	
EmuJ_000849600	Proteinase inhibitor I25 cystatin	1.7	±	0.8	
EmuJ_000703300	Actin	1.1	±	0.6	
EmuJ_000579800	Histone H3	1.0	±	0.8	
EmuJ_000388100	Histone H4	1.0	±	1.0	
EmuJ_001201500	Histone H2A	0.9	±	0.6	
EmuJ_000908900	N acetylated alpha linked acidic dipeptidase 2	0.8	±	0.4	
EmuJ_000641100	Alpha 2 macroglobulin	0.5	±	0.2	
EmuJ_000724500	Expressed protein	0.5	±	0.3	
EmuJ_000383700	Histone H2B	0.4	±	0.3	
EmuJ_000036300	Actin cytoplasmic type 5	0.3	±	0.1	
EmuJ_000682900	Niemann Pick C2 protein	0.3	±	0.2	
EmuJ_000184900	Glycoprotein antigen 5	0.3	±	0.1	
EmuJ_000085400	Mastin	0.2	±	0.1	
EmuJ_000905600	Fructose-bisphosphate aldolase	0.2	±	0.1	

The complete dataset is presented in **Table S4**. Mean values ± standard deviations correspond to five biological replicates.

### 3.2 Immunofluorescence

All five antisera against the different AgB polypeptides, including the pre-immune sera, were characterized regarding their reactivity with the pure peptides AgB8/1–AgB8/5 and *in vitro* generated VF (**Figure S1**). The detection limit of AgB8 polypeptide antisera with specific AgB8 polypeptides was 2 ng in all cases. For VF, the detection limit was at a dilution of 1:64 or pure in the case of anti-AgB8/5. No detectable cross-reactivity with other AgB8 polypeptides was observed at 125 ng, except in the case of anti-AgB8/2, which faintly cross-reacted with AgB8/5 at 125 ng. The preimmune sera cross-reacted with pure VF only and not with any of the pure AgB polypeptides.

**Figure 2:**
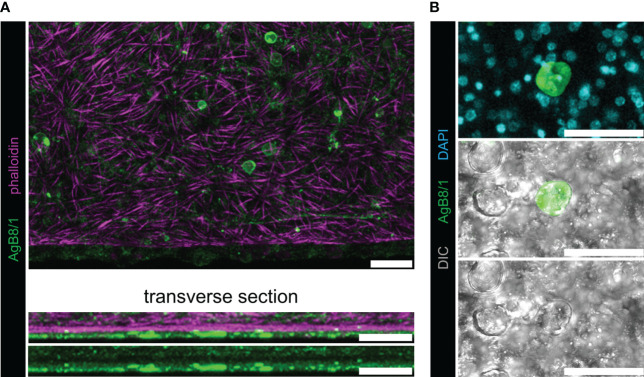
Whole-mount immunofluorescence with polyclonal antibodies against AgB8/1 polypeptide. With all other AgB antisera, similar results were obtained (**Figure S2**), and representative pictures of AgB8/1 labelings are shown. No signal was detected with preimmune sera (**Figure S3**). **(A)** *E. multilocularis* metacestode grown *in vitro* labeled with anti-AgB8/1 (green) and rhodamine-phalloidin (magenta). The upper panel shows maximum intensity projection and lower panels show transverse sections. **(B)** Maximum intensity projection with higher magnification showing a calcareous corpuscle stained with anti-AgB8/1 and nuclei (DAPI, cyan) in the upper panel, with DIC (differential interference contrast, gray) in the lower panels (note that not all calcareous corpuscles were stained). Scale bars: 30 µm.

Applying polyclonal antisera generated for each individual AgB polypeptide, we detected a strong signal located beneath the phalloidin-stained subtegumental muscle layer that surrounds the *E. multilocularis* metacestode. This signal was exclusively present in some calcareous corpuscles of *E. multilocularis* metacestodes (**Figure 2**; **Figures S2**, **S3**). The signal co-localized to the calcareous corpuscles that were identified by bright-field imaging and by the presence of eccentric nuclei (Koziol et al., 2014).

### 3.3 Uptake of a labeled AgB subunit analyzed by targeted mass spectrometry

**Figure 3:**
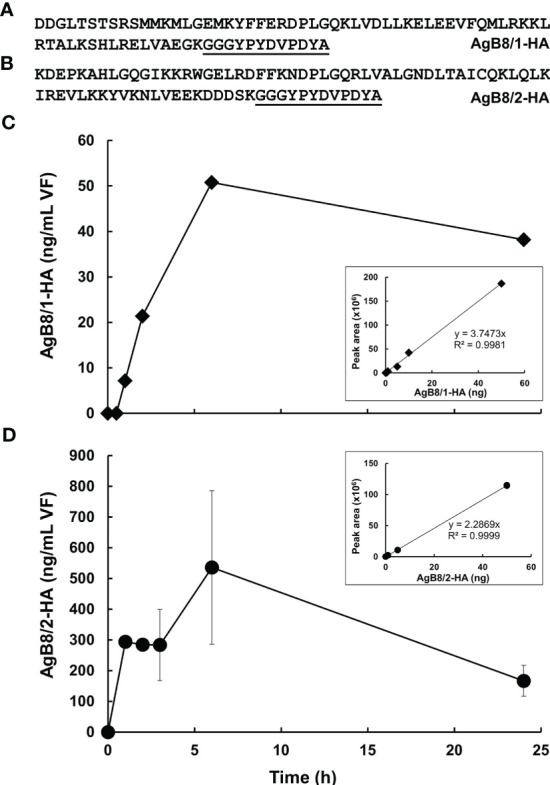
Uptake of HA-tagged AgB subunits into *E multilocularis* metacestodes. **(A)** Polypeptide sequence of HA-tagged AgB8/1 encoded by EmuJ_000381200. **(B)** Polypeptide sequence HA-tagged AgB8/2 encoded by EmuJ_000381100. HA-tag is underlined. **(C)** Pre-study uptake with HA-tagged AgB8/1. **(D)** Uptake study with HA-tagged AgB8/2. Mean values ± standard errors correspond to three replicates. The calibration curves of the corresponding HA-tagged polypeptides are presented as inlets. Culture medium was spiked with HA-tagged polypeptide, vesicle fluid was harvested, and samples were processed as described in Materials and Methods. The full dataset is presented in **Tables S5**, **S6**.

The observation that AgB subunits were detected in CM and previous suggestions in the literature prompted us to investigate whether a re-uptake of AgB occurred. For this purpose, we selected the AgB8/1 subunit EmuJ_000381200 and the AgB8/2 subunit EmuJ_00038100, the second and third most abundant subunits in VF, VF *ex vivo* and CM, and offered the corresponding artificial polypeptide decorated with an HA-tag at its C-terminus (**Figures 3A, B**) to *E. multilocularis* metacestodes in CM. A pre-experiment was performed with AgB8/1. To establish a suitable quantification method, VF prepared as described above was spiked with increasing amounts of the HA-tagged polypeptide. By targeted mass spectrometry, peptides matching the native AgB8/1 subunit EmuJ_000381200 as well as the HA-tagged peptide could be detected. The HA-tag was, however, detectable in the spiked VF only (**Table S5**). The signal strength corresponding to this tag was proportional from 0 to 50 ng of spiked polypeptide. Then, metacestodes were incubated in CM containing the HA-tagged AgB8/1 subunit EmuJ_000381200, and VF was harvested at various time points. Within 6 h, the amount of HA-tagged AgB8/1 in VF increased from 0 to ca. 50 ng/ml, followed by a decrease (**Figure 3C**). This concentration was, however, three orders of magnitude lower than the initial concentration in the medium, namely, 50 µg/ml (**Figure 3C**). Based on these results, we performed a second, larger scale experiment with peptide AgB8/2 under the same conditions. Again, the amount of HA-tagged peptide increased within 6 h, to 535 ng/ml (± 250), followed by a drop (**Figure 3D**; **Table S6**).

## 4 Discussion

Our results have revealed that AgB subunits are the most prominent parasite proteins in vesicle fluid (VF) and CM of *in vitro* cultured *E. multilocularis* metacestodes. Their pattern is even conserved in VF *ex vivo*. In all samples, the most prominent subunit is the gene product of EmuJ_000381500, previously annotated as the isoform AgB8/3a. EmuJ_000381800 (AgB8/5) is the only AgB subunit not within the top 20 proteins of any of the samples.

In a previous study, the proteomic composition of *E. mutlilocularis* metacestode vesicles cultured *in vitro* was analyzed, and interestingly, this included one of the parasite isolates applied in the here presented study, H95 (Monteiro et al., 2017). The authors analyzed samples by LC-MS/MS and detected substantially fewer parasite proteins than in the present study, which shows that the proteomic methodology was less sensitive. They detected AgB subunits that followed the following abundance: EmuJ_000381500 > 600 > 200 > 100. As in our study, EmuJ_000381800 was not detected in the H95 isolate. The high abundance of EmuJ_000381500 is in excellent agreement with the present study. However, Monteiro et al. annotated this subunit as AgB8/5, whereas EmuJ_000381500 is annotated here and in Ahn et al. (2017b) as AgB8/3a. In the present study, but not in the previous study published by Monteiro et al. (2017), the subunit EmAgB8/4 was detectable in *in vitro* cultured *E. multilocularis* metacestodes of the isolate H95. Also, Monteiro et al. (2017) detected EmuJ_000381600 as the second most abundant subunit, which is not in line with our findings. Apart from that, the same order of subunits was detected in both studies.

In another study, VF was collected from Kunming mice infected with *E. multilocularis* and proteomic analysis was performed based on 2 DE and MALDI-TOF (Ahn et al., 2017b). This methodology had low sensitivity and included semi-quantitative analyses based on spot intensity. The *ex vivo* analysis by Ahn and colleagues allowed the detection of all AgB subunits except EmAgB8/5. EmAgB8/3a was the most prominent subunit, followed by EmAgB8/4, EmAgB8/2, EmAgB8/3b/c, and EmAgB8/1. In the same study, further analyses on the transcriptional level with actin as control showed the following relative expression pattern: EmAgB8/2 > EmAgB8/4 > EmAgB8/3b/c > EmAgB8/3a > EmAgB8/5 > EmAgB8/1. This transcriptomic analysis of AgB subunit expression was contradicted by the RNASeq study conducted by Huang et al. (2016), who found the following expression pattern in *ex vivo* harvested metacestodes from infected DBA/2 mice: EmAgB8/3a > EmAgB8/1 > EmAgB8/4 > EmAgB8/3b/c > EmAgB8/2 (transcripts for EmAgB8/5 were not detected). In the same study, also *in vitro* generated *E. multilocularis* metacestodes were analyzed by RNASeq, and a very similar pattern was found: EmAgB8/3a > EmAgB8/1 > EmAgB8/4 > EmAgB8/2 > EmAgB8/3b/c. Transcripts for EmAgB8/5 were not detected (Huang et al., 2016). Stage-specific expression of EmAgB subunits was also investigated by Mamuti et al. (2006). By RT-PCR, the authors found metacestode vesicles to express all AgB subunits except EmAgB8/5, and EmAgB/1 was expressed the highest, followed by EmAgB8/3 and EmAgB8/2, and EmAgB8/4 was expressed the lowest. They confirmed the expression of these four subunits by Western blot, but no quantitative analyses were performed.

Summarizing, EmAgB8/3a is the most abundantly expressed AgB subunit in the proteomic analyses of Monteiro et al. (2017) and Ahn et al. (2017b), in the transcriptomic analyses by Huang et al. (2016), and in our present study. It is to be noted that previous studies on the distribution of EgAgB subunits in hydatid fluid from *E. granulosus s.l.* have all identified the subunit EgAgB8/1 to be the most abundant subunit (Monteiro et al., 2012; Silva-Alvarez et al., 2016; Folle et al., 2017). Thus, there are clear differences in the distribution of AgB subunits between the *Echinococcus* species.

The second most abundant AgB subunit we detected is EmAgB8/1, which is also the second most abundant subunit in the transcriptomic analyses by Huang et al. (2016). Monteiro et al. (2017) detected EmuJ_000381600 (here annotated as EmAgB8/3b) as the second most abundant subunit. In the study by Ahn et al. (2017b), EmAgB8/4 is the second most abundantly detected AgB subunit. Also for *E. granulosus*, EgAgB8/4 was the second most abundantly detected subunit (Monteiro et al., 2012; Silva-Alvarez et al., 2016; Folle et al., 2017). All other subunits follow different patterns from Ahn et al. (2017b) or Huang et al. (2016), both in our *in vitro* generated samples and in the metacestodes harvested from infected BALB/c.

In contrast to previous studies (Ahn et al., 2017b; Ahn et al., 2017c), EmAgB5 is clearly detected in our study in VF *in vitro* and *ex vivo*, but only in negligible amounts as compared to the other subunits. Also, Folle et al. (2017) detected AgB8/5 in hydatid fluid of *E. granulosus s.l.* as the least abundant subunit.

For all the here-described comparisons to other studies, it should be noted that in particular for *ex vivo* samples, other factors such as parasite isolate, cyst viability, location, or host species and/or strains might be an uncontrollable source of variation. For this reason, this study focused on a standardized *in vitro* model. Moreover, comparisons of pool-size analyses like the proteomic composition of VF can only be limitedly compared to transcriptomic data, which were generated based on RNA levels in GL cells.

The observation that AgB subunits are excreted to the CM is in line with their immunomodulatory role and their use in diagnostics of echinococcosis (Siracusano et al., 2008a). A prerequisite for this function is contact with the host immune system and thus excretion from the cyst to the host and uptake into immunomodulatory host cells (Silva-Alvarez et al., 2016). Besides their immunomodulatory role, the AgB lipoproteins may transport lipids from the host to the parasite and therefore exert metabolic functions (Silva-Alvarez et al., 2015a). Similarly, the apolipoprotein binding protein of *E. multilocularis* is suggested to be involved in the uptake of host lipids and cholesterol (Bernthaler et al., 2009). Moreover, the lipid uptake in another cestode, *Taenia solium*, may be mediated by a hydrophobic lipid-binding protein (Lee et al., 2007). If AgB subunits are involved in lipid uptake, a secretion into and re-uptake from the medium into the metacestode vesicle is necessary. Our experiments with an HA-labeled AgB subunit show that such a re-uptake may occur. Both peptides AgB8/1-HA and AgB8/2-HA (representatives of the two different AgB subfamilies) are taken up time-dependently, albeit uptake of AgB8/2-HA appeared to be more efficient. Further studies on uptake rates will have to be performed to confirm real differences in uptake rates and selectivity compared to control proteins. Since the highest concentration in the VF is far from the equilibrium, passive diffusion along the concentration gradient between CM and VF is unlikely to occur. Rather, the subunits are taken up by endocytosis, or possibly specific transporters, into the GL followed by exocytosis into the VF. The high level of the endophilin B1 (Takahashi et al., 2009) homologous protein p29 (EmuJ_000550800) in the VT could be indicative of “fast endophilin mediated endocytosis” (Casamento and Boucrot, 2020) as responsible for this process. Interestingly, the three most abundant proteins in VT might be involved in excretory/secretory processes: the AgB subunit is an excretory/secretory product, the two others, the expressed conserved protein encoded by EmuJ_000412500 and the protein p29, might be components of the machinery responsible for this process, as suggested by homology to proteins in other eukaryotes. The presence of host proteins within the VF, as shown by others (Monteiro et al., 2017), indicates that proteins from various origins are taken up. It has to be elucidated whether this uptake occurs non-specifically or whether distinct transport systems for different proteins exist. One potential function of this process could be the transfer of lipids or other nutrients from the host to the metacestode; another function could be the resorption (and thereby inactivation) of components of the host immune system from the surface of the metacestode. Interestingly, *E. granulosus s.l.* AgB interacts with host C-reactive protein, as evidenced in a previous study (Silva-Alvarez et al., 2018).

All AgB subunits localized to the surface of the calcareous corpuscles in the metacestodes, as observed previously in cysts *ex vivo* from infected rats (Rickard et al., 1977). Fasciclin, another calcareous corpuscle-associated protein, was shown to ensure survival of the cestode *Taenia solium* in the host (Ahn et al., 2017a). It is important to note that the here-described antisera against the five polypeptides of AgB might be cross-reacting due to high antigen sequence homology, and thus, no individual interpretations can be made, even though our analyses did reveal only cross-reactivity between the AgB8/2 antiserum and the AgB8/5 polypeptide.

Apart from nutrient acquisition or resorption and inactivation of host immune components, a third function of protein uptake, including the unspecific uptake of host proteins, could reside in the maintenance of a low water potential within the metacestode. In fact, metacestodes are liquid-filled vesicles surrounded by host tissue. Since this tissue exerts a pressure on the metacestode surface, the metacestode would collapse or burst. To prevent this, an internal counterforce maintaining the vesicular shape is needed. This force is derived from the water potential inside the vesicle causing an influx of water from the host tissue (or from the CM) thereby creating an internal pressure against the tegument, a “turgor”. This water potential has two components, namely, an osmotic component proportional to the amount of non-diffusible solutes within the VF and a matric component proportional to the amount of water binding macromolecules such as proteins. Since macromolecules are in general less diffusible than micromolecules and thus better retained within the lumen of the metacestode, the water potential of the VF could largely depend on its matric component. During the growth of the metacestodes, the intake of proteins (or other active macromolecules) increasing the influx of watershould occur in parallel to the enlargement of the surface, i.e., extension of GL and LL. To ensure a low matric water potential, the proteins should, however, remain intact after their uptake. The presence of an alpha 2 macroglobulin homolog (EmuJ_000641100), thus a potential proteinase inhibitor (Rehman et al., 2013), as one of the most prominent proteins both in VF and in the CM, could preserve the integrity of proteins before and after uptake, thereby acting as a safeguard for this mechanism.

Taken together, standardized tools are paramount to understand the development of any biological system. The fact that our data obtained from VF *in vitro* are in good agreement with *ex vivo* data underlines the validity of our model. It supports the analysis of protein excretion from and uptake into the *E. multilocularis* metacestode, thereby paving the way for further experiments including labeling of potential ligands providing deeper insights into the biology of this helminth.

## Data availability statement

The datasets presented in this study can be found in online repositories. The names of the repository/repositories and accession number(s) can be found in the article/**Supplementary Material**.

## Ethics statement

The animal study was reviewed and approved by Veterinäramt Kanton Bern.

## Author contributions

JM, MP, MH, and BL-S contributed to conception and design of the study. RR and BL-S performed the sampling. SB, A-CU, and MH analyzed the proteomic samples. JM, MP, SB, A-CU, MH, and BLS performed data analysis. MP performed the immunolocalization and sequence analyses. MK conducted the *in vitro* uptake experiment. JM, MP, and BL-S drafted the manuscript. Allauthors contributed to the article and approved the submitted version.

## Supplementary material

Supplementary Figure 1 Validation of anti-AgB8-antisera with dot-blots. **(A)** Example of dot-blot testing of anti-AgB8/1 serum and AgB polypeptides for cross-reactivity. In addition, reactivity with VF was tested. **(B)** Summary of results of all respective dot-blots for the five different AgB polypeptides and antisera. CR, cross-reactivity; DL, detection limit, nd, not detectable at 125 ng; VF, vesicle fluid.

Supplementary Figure 2 Whole mount immunofluorescence with polyclonal antisera against the different AgB polypeptides. Metacestode grown *in vitro* with antibodies against AgB8/2 **(A)**, AgB8/3 **(B)**, AgB8/4 **(C)**, AgB8/5 **(D)** as visualized in green. In merge with nuclear DAPI staining in cyan and DIC (differential interference contrast) in grey. Note that the upper panels show the overview at lower magnification, and the lower panels show higher magnification of labeled calcareous corpuscles. Scale bars: 30 µm.

Supplementary Figure 3 Negative controls for the whole mount immunofluorescence with preimmune sera. Metacestodes grown *in vitro* were stained with preimmune sera from rabbits used for generation of the antisera against AgB8/1 **(A)**, AgB8/2 **(B)**, AgB8/3 **(C)**, AgB8/4 **(D)** and AgB8/5 **(E)**, and detected with a secondary anti-rabbit-FITC antibody (green). In merge with nuclear DAPI staining in cyan and DIC (differential interference contrast) in grey. Scale bars: 30 µm.

Supplementary Table 1 Polypeptide sequences that were synthesized and used for antiserum generation in rabbits.

Supplementary Table 2 Data file from LC-MS/MS analyses for all peptides identified in VF and VT of *in vitro* grown *E. multilocularis* metacestodes.

Supplementary Table 3 Data file from LC-MS/MS analyses for all peptides identified in CM of *in vitro* grown *E. multilocularis* metacestodes.

Supplementary Table 4 Data file from LC-MS/MS analyses for all peptides identified in VF of *E. multilocularis* metacestodes harvested from infected mice.

Supplementary Table 5 Full dataset of AgB8/1-HA uptake experiment.

Supplementary Table 6 Full dataset of AgB8/2-HA uptake experiment.
