# Supplementary figures and images for: Targeted and non-targeted proteomics to characterize the parasite proteins of Echinococcus multilocularis metacestodes
Source: Front Cell Infect Microbiol. 2023 May 30;13:1170763. doi: 10.3389/fcimb.2023.1170763 (PMC10266102; doi:10.3389/fcimb.2023.1170763)

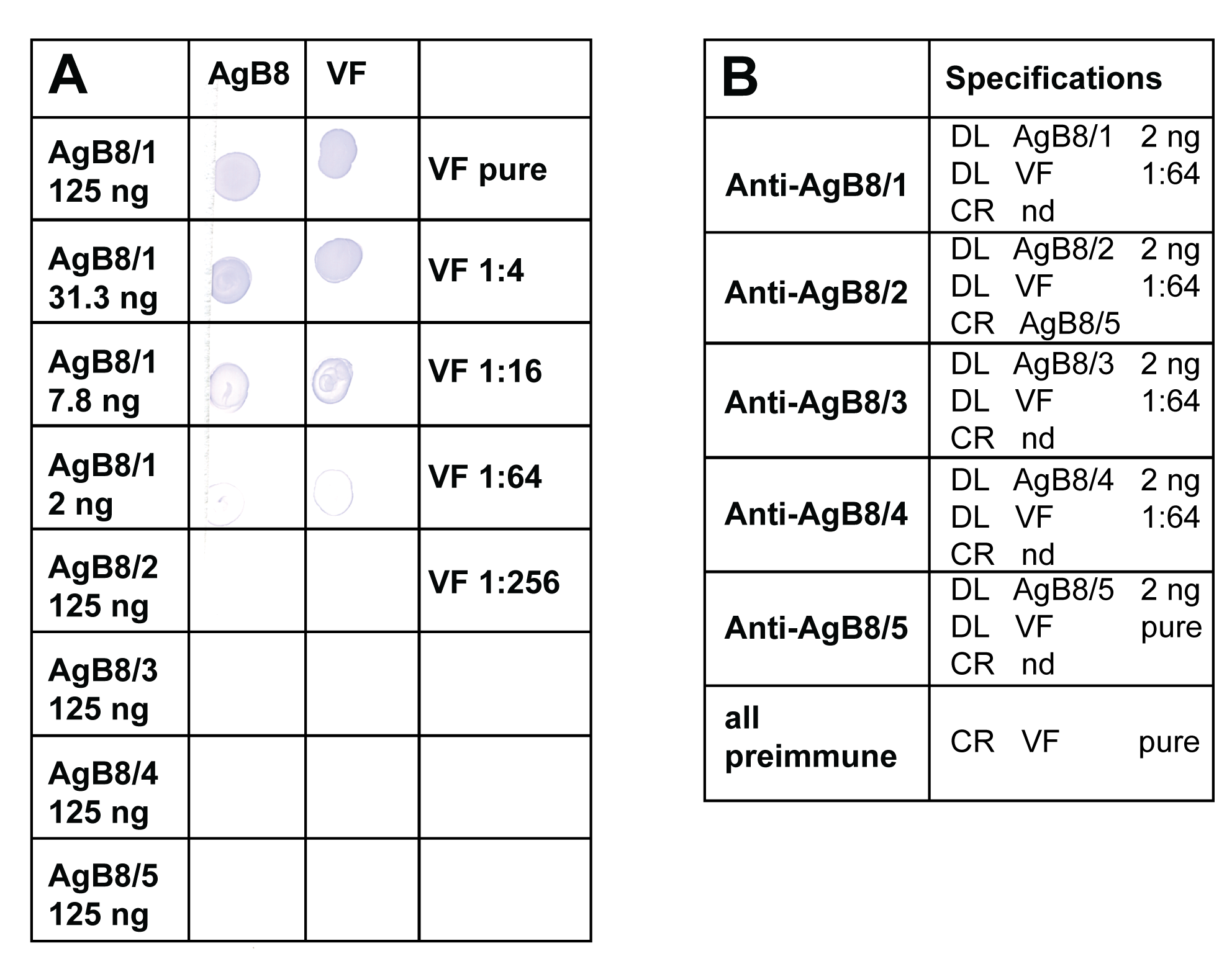

Supplement: Supplementary Figure 1 — Validation of anti-AgB8-antisera with dot-blots. (A) Example of dot-blot testing of anti-AgB8/1 serum and AgB polypeptides for cross-reactivity. In addition, reactivity with VF was tested. (B) Summary of results of all respective dot-blots for the five different AgB polypeptides and antisera. CR, cross-reactivity; DL, detection limit, nd, not detectable at 125 ng; VF, vesicle fluid. [file Image_1.tif]

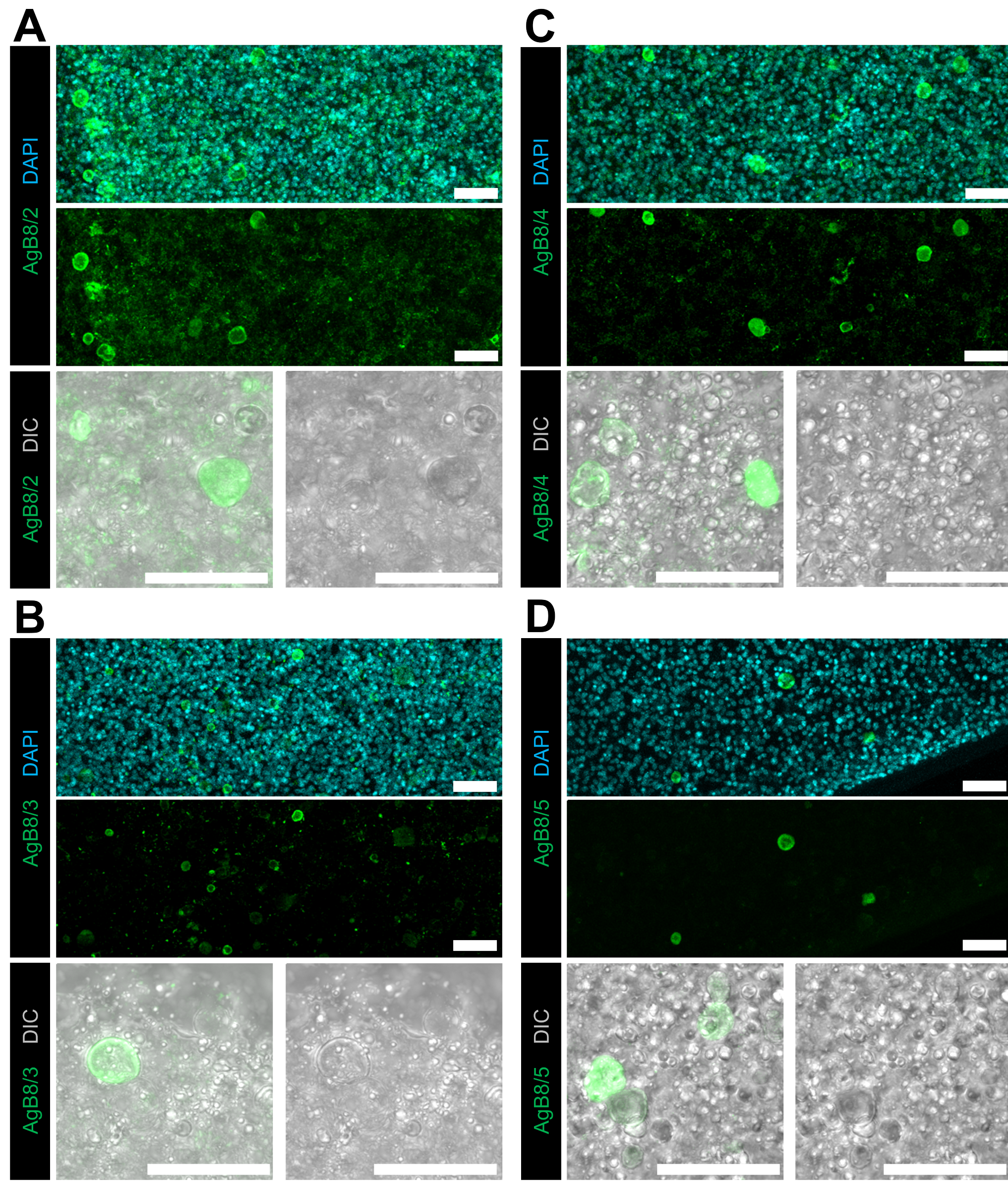

Supplement: Supplementary Figure 2 — Whole mount immunofluorescence with polyclonal antisera against the different AgB polypeptides. Metacestode grown in vitro with antibodies against AgB8/2 (A), AgB8/3 (B), AgB8/4 (C), AgB8/5 (D) as visualized in green. In merge with nuclear DAPI staining in cyan and DIC (differential interference contrast) in grey. Note that the upper panels show the overview at lower magnification, and the lower panels show higher magnification of labeled calcareous corpuscles. Scale bars: 30 µm. [file Image_2.tif]

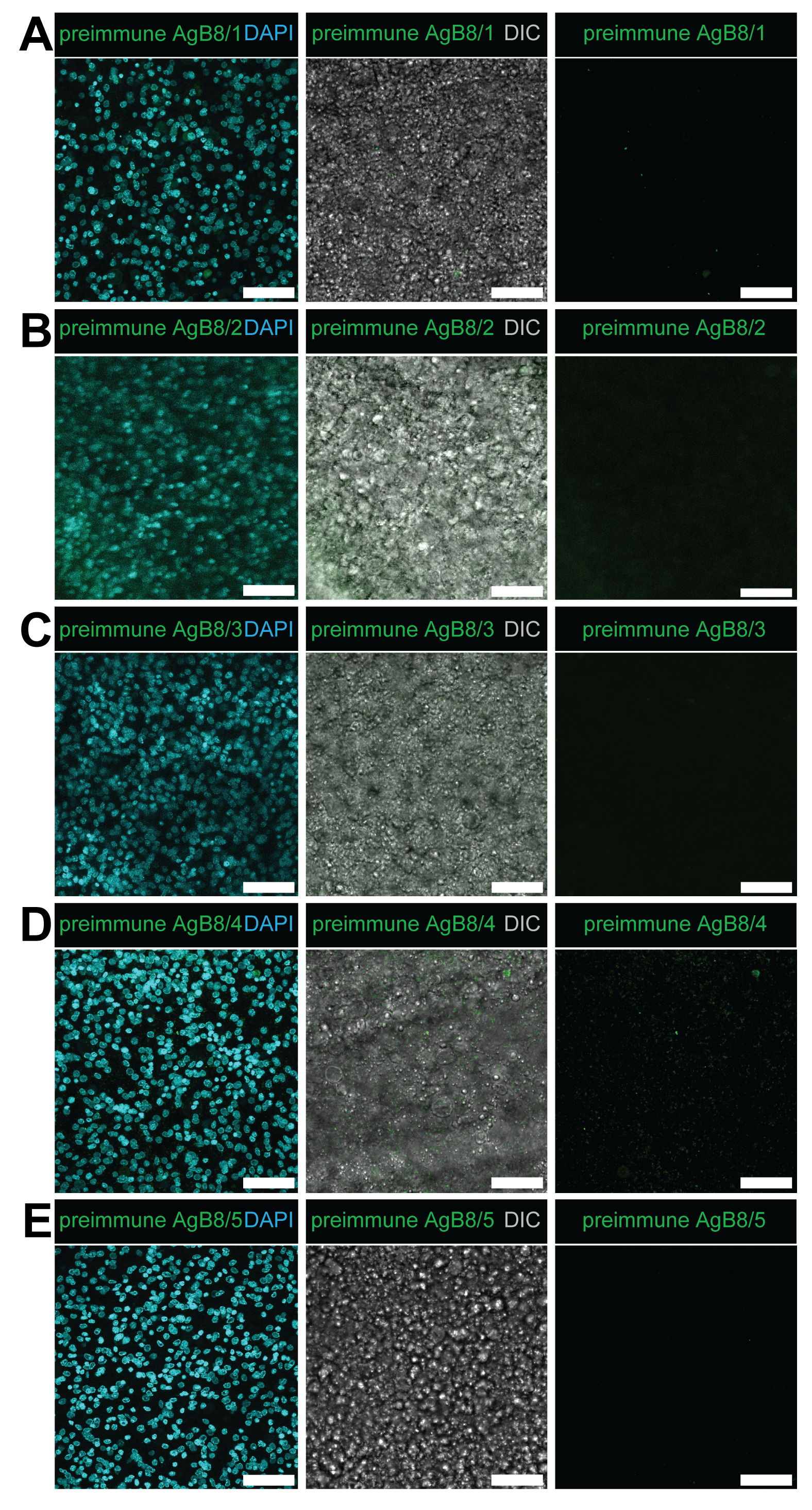

Supplement: Supplementary Figure 3 — Negative controls for the whole mount immunofluorescence with preimmune sera. Metacestodes grown in vitro were stained with preimmune sera from rabbits used for generation of the antisera against AgB8/1 (A), AgB8/2 (B), AgB8/3 (C), AgB8/4 (D) and AgB8/5 (E), and detected with a secondary anti-rabbit-FITC antibody (green). In merge with nuclear DAPI staining in cyan and DIC (differential interference contrast) in grey. Scale bars: 30 µm. [file Image_3.tif]
